# Exploring the motivations behind misreporting self-measured blood glucose in adolescents with type 1 diabetes – a qualitative study

**DOI:** 10.1186/s40200-016-0238-6

**Published:** 2016-06-04

**Authors:** Miranda Blackwell, Paul A. Tomlinson, Jenny Rayns, Jackie Hunter, Annika Sjoeholm, Benjamin J. Wheeler

**Affiliations:** 1 Department of Women’s and Children’s Health, University of Otago, PO Box 913, Dunedin, 9054 New Zealand; 2 Department of Paediatrics, Southern District Health Board, Invercargill, New Zealand; 3 Paediatric Endocrinology, Southern District Health Board, Dunedin, New Zealand; 4 Department of Psychology, University of Otago, Dunedin, New Zealand

**Keywords:** Type 1 diabetes mellitus, Adolescent, Self-monitoring blood glucose, Non-adherence, Misreporting

## Abstract

**Background:**

Despite advances in diabetes management, the reporting and self-monitoring of blood glucose (SMBG) remains fundamental. While previous work has established that the misreporting of SMBG to family and medical professionals is surprisingly common, the motivations behind this behaviour have never been examined. We aimed to investigate the motivations behind misreporting of SMBG in adolescents with type 1 diabetes (T1DM).

**Methods:**

Fifteen semi-structured interviews were conducted with adolescents (aged 12–19 inclusive) with T1DM recruited through diabetes clinics across the Otago/Southland region of New Zealand from November 2015 to January 2016. These were transcribed and content analysis performed to identify themes and subthemes in misreporting behaviour.

**Results:**

The mean age of participants was 15.7 years, 60 % were male, with 67 % using multiple daily insulin injections, and 33 % on insulin pumps. Their median HbA1c was 84 mmol/mol, range 52–130. Misreporting behaviour was described for both electronic pump records and written logbooks, as well as verbally. Multiple motivations for misreporting were given, spanning three major themes: Achieving potential benefits; the avoidance of negative consequences; and the avoidance of worry/concern (in self or in others). The main suggestion of participants to reduce misreporting behaviour was to reduce the negative reactions of others to suboptimal blood glucose readings.

**Conclusion:**

Electronic, written, and verbal SMBG misreporting remains common. This study provides deeper insight into the motivations leading to this behaviour in adolescents, suggesting that further understanding and attention to this aspect of adherence may lead to improvements not only in glycaemic control and safety, but also to the psychological wellbeing of those with T1DM.

## Background

Despite considerable advances in insulin delivery and glucose monitoring technology, self-monitored blood glucose (SMBG) remains a vital component of modern type 1 diabetes mellitus (T1DM) management. SMBG is not only essential for day to day monitoring, safety, and dose adjustment, it is also associated with improved glycaemic control, with suggested improvements in HbA1c of up to 0.5 % (5.5 mmol/mol) with each additional SMBG up to a maximum of 5-6/day [1, 2]. This positive impact on glycaemic control is likely to be multifactorial, potentially reflecting the benefits of a more intensive insulin regimen, and of improved diabetes self-care/self-determination [2]. However, SMBG frequency is also likely to be a surrogate marker for overall adherence (or not) with ones diabetes management.

Non-adherence with SMBG can occur in a number of different ways. The simplest form is a reduced frequency in SMBG. Misreporting of SMBG to parents/caregivers and health professionals represents another more complex aspect. This can take various forms, including: verbal misreporting, usually between a child and their parents (but occasionally health professionals); and logbook misreporting, both written and electronic, which includes manipulating pump download data. Misreporting also has both acute and chronic dangers, including both hypo- and hyperglycaemia. Considering how common, and important SMBG is to health and safety in T1DM, there are few studies examining misreporting, particularly in children.

The first study conducted compared SMBG written logbook entries to meter memory in 19 adults over a two week time period. They found that 75 % misreported their blood glucose level (BGL) over the course of the study, mostly by reporting a lower BGL than actually recorded in order to appear more favourable. Efforts to conceal hypoglycaemia were also seen [3]. Subsequent logbook studies have supported these findings, with 47 % to 55 % having some form of discrepancy between their SMBG and logbook entries [4–6]. Fewer studies have been conducted in children and adolescents, but the findings appear consistent [7]. In addition, two studies have looked at the accuracy of verbally reported SMBG in adolescents. In the first, 70 % of the study population verbally misreported SMBG to their camp supervisors, with an overall error rate of 13.5 % [8]. In a follow up study, by making adolescents aware of SMBG surveillance, this misreporting rate fell to 4.7 % [9]. Therefore, while some investigation of the rates of misreporting has occurred, no data exploring the motivations underlying this important behaviour are available. In addition, investigation of misreporting in the modern pump era is also missing.

Understanding what motivates this behaviour may lead to opportunities to decrease its impact, as well as provide increased support to people struggling with this complex illness. This seems particularly relevant in the adolescent age group, a time of significant developmental, social and behavioural change, as well as a time where non-adherence in general is particularly prevalent [10]. With these factors in mind, this study aimed to investigate the motivations behind misreporting of blood glucose levels in adolescents with T1DM.

## Methods

### Study design

Fifteen semi-structured interviews were used to investigate the motivations behind misreporting of SMBG in teenagers, aged 12–19 years (inclusive) with T1DM. The sampling strategy involved the consecutive approach of appropriate diabetes clinic patients, over two clinical sites. To give an indication of uptake of this study, in total 29 patients were approached, giving a total recruitment rate of 52 %. This final sample size was determined by the presence of relative data saturation, in that, for interviews 13-15 no new information/perspectives in relation to themes or sub-themes were added, other than reinforcing those previously described.

In preparation for the study, an interview guide was developed through literature review, and discussions with representatives from paediatric endocrinology, diabetes specialist nursing, and psychology. Interviews included: basic demographic and diabetes data including age, prioritised ethnicity, management details, and socioeconomic status (using the established and previously reported NZ Deprivation index [11]); feelings about T1DM; their history and methods of misreporting; and motivations behind misreporting.

### Study setting and participants

The study was conducted from November 2015 to January 2016, in the Otago/Southland region of New Zealand. Participants were recruited during diabetes clinics, which provide care to all those with diabetes in this region, approximately 160 children. The key inclusion criteria were: 1) T1DM with a duration of ≥ 6 months, and on >0.5 units of insulin/kg/day; 2) aged 12 – 19 years; 3) adequate English, or availability of an interpreter for the study interview; 4) and a willingness to admit/discuss misreporting behaviour. In addition, three potential participants were excluded as their treating team felt the study would not be appropriate given a current acute psychosocial morbidity.

### Data collection and analysis

Once written informed consent was obtained, interviews were carried out by an investigator not involved with usual diabetes care (MB/AS for the first two, then MB alone). This was in order to avoid participant perception of any potential negative consequence. For the same reasons, parents/caregivers were also not present. In addition, participants were made aware that the interviews were confidential, with no identifiable content to be reported back to either their treatment team, nor their families.

Interview location varied, with 10 occurring in hospital directly following diabetes clinic, and five in the participant’s home. Interviews lasted between 20 and 40 min (median 24 min). All interviews were digitally recorded and transcribed, and then organised using a coding system that reflected the interview guide as well as commonalities noted during analysis. Thematic analysis followed, with coded segments of data sorted into themes through an iterative process of discussion between the research team. Identification of a theme was not necessarily dependent on frequency of report, but rather whether it represented an idea that captured an important aspect of misreporting.

## Results

Basic demographic information of participants is displayed in Table 1.

**Table 1:** Basic demographic information of study population

	Total Sample (*n* = 15)
Male Sex; n (%)	9 (60 %)
Mean Age; yr (SD)	15.7 (2.0)
Mean age at diagnosis; yr (SD)	9.1 (3.2)
Insulin regimen: pump/MDI; n (%)	5 (33 %)/10 (67 %)
Median latest HbA1c; mmol/mol (range)	84 (52–130)
Median Interview Duration; minutes (range)	24:10 (11:03–46:53)
Ethnicity: NZ European; n (%)	13 (87 %)
Ethnicity: Māori; n (%)	2 (13 %)
Median NZDep2006^a^ Index (range)	5 (2–9)

*MDI* multiple daily injections

^a^NZDep2006 Deprivation Index: a measure of socioeconomic status [10]

Participants described efforts to misreport to parents, wider family, health professionals, and others, such as teachers and friends. Methods of misreporting included written logbook, verbal, and efforts to fabricate meter/pump download data. Pump users only used the latter two methods. Fabrication of meter/pump data was described in several ways, including: inputting inaccurate data into the pump, usually in the form of a phantom (not actually measured) or a lower, or more “normal” BGL than measured; another method used is demonstrated by the following quote:

“I tested my friend’s blood on my thing so it looked like I was really good” (participant 13).

Three major themes emerged regarding motivations for misreporting, including: Achieving potential benefits; the avoidance of negative consequences; and the avoidance of worry/concern, in self or in others. These themes, including related sub-themes with representative quotes are summarised in Table 2.

**Table 2:** Representative quotes

Theme (*n*)^a^	Quote (participant number)
Achieving benefits *(14/15)*	
Gaining food *(7/15)*	1) “Whenever you feel hungry in class you just say you’re low so you can get something to eat.” (7)
Being excused from school activities *(4/15)*	2) “One time, we were in a test and I said I was low so I didn’t have to do it” (15)
Being allowed to continue doing something *(10/15)*	3) “Last week I told them that it was (lower than it was) because I’d just woke up and they (family) told me to test and I wanted to go back to sleep so I tested, it was 26 or something, and I went out and said “I’m good it’s 5.2. I’m going back to bed.” (3)
Avoidance of negative consequences *(14/15)*	
Avoiding adult censure *(12/15)*	4) “So before tea she (mum) used to ask me all the time “what was your reading?” If I told her it was high she’d be angry, so I just told her a good number…” (8) 5) “Making sure that Dr X’s not going to yell at you and just to make him happy. If I can’t remember a reading I’ll put it down a bit so it looks better when I go to the hospital for a check-up.” (14)
Maintenance of autonomy *(11/15)*	6) “Gets a bit irritating if they (parents) don’t know what they’re doing. They try to tell you different things but it doesn’t help… I just tell them a good number like “this is what it is leave me alone.” (8) 7) “If mum randomly asked what my level was I’d just say “oh 10” when it’d be 20.”…” Otherwise she’d be nagging and annoying me.” (12)
Avoiding coming to clinic *(1/15)*	8) “I didn’t like coming to clinic, so I thought if I lied I wouldn’t have to come back for like 6 months…” (12)
Avoiding embarrassment or exclusion *(10/15)*	9) “I used to (not test) because people used to be like “Ew what are you doing?” or want to watch and it made me feel like shit so I didn’t do it around other people.… I’d go home and write down in my book that I was 8 at lunchtime.” (12) 10) “I lied at diabetes camp once. Because they did this thing where you call out what your blood sugar was and mine wasn’t too good so I just said it was 6.5. Then they did the ketones and I lied about my ketones as well… everyone else’s blood sugar was good and I was sad… I was really embarrassed. So I said “No my blood sugar’s fine” just to fit in… I looked up to all the people who had really good control.” (6)
Fear of missing out *(10/15)*	11) “If I was with friends or whatever and I didn’t want to test because we were having fun I wouldn’t stop to do a test- I’d just put down 8.” (12) 12) “If I’m in a rush, I may not test, I might just put a random number in.” (1)
Avoidance of worry/concern in others or self *(14/15)*	
Avoiding emotional distress in others *(11/15)*	13) “I just don’t tell them (parents) how bad it is so they don’t worry about me. I can deal with it myself without people nagging me. Probably that. I’m independent and no one needs to be concerned about me.” (14) 14) “I’d spend about 3 h writing in the logbook full of random numbers. I wasn’t a bad kid I just felt like I was letting the doctors down. They’d put so much time and effort into me and I felt bad that I wasn’t looking after myself.” (11) 15) “She (mum) wanted to know what they (blood sugars) were and I knew that they weren’t going to be what she wanted so I just said what she wanted to hear.” (13)
Avoiding emotional distress in self *(8/15)*	16) “Maybe about a month or so ago I was on the computer all day and my blood sugar just wasn’t changing. My dad kept coming in and asking me what it was and I kept telling him, and he got more frustrated and more annoyed so I just finally lied to him and said “no it’s fine” because I was so annoyed with my blood sugars and that they weren’t going down. It wasn’t really my dad it was just me being annoyed.” (6)

^a^ *n* number of participants describing each theme/subtheme

1. Achieving potential benefits
  Under this category fell instances of misreporting where the participants perceived a potential benefit, such as: accessing food; an excuse to avoid something unpleasant; or continue an activity uninterrupted. The most common of these (reported by the majority), being the fabrication of hypoglycaemia in order to get food, especially during school (quote 1). Similarly, participants also reported factitious hypoglycaemia in order to avoid school classes or assessment (quote 2), and to access longer lunch breaks.

2) Avoiding negative consequences
  This included misreporting to avoid perceived negative situations, such as avoiding adult censure, being interfered with or bothered, coming to clinic, embarrassment or missing out on something. The most common scenario reported (12/15) was when participants anticipated their measured SMBG would receive a negative reaction or response (from parents or treatment team). In an effort to avoid this, misreporting to make the number appear more favourable usually resulted. This occurred both verbally to parents (quote 4), and electronically or in written logbooks for parents and diabetes team (quote 5).
  Efforts to avoid interference from others was a related theme. Participants frequently reported concealing hyperglycaemia to avoid having to explain diabetes to people such as parents or teachers who they perceived as misunderstanding it. Many participants viewed themselves as being independent enough to deal with their blood glucose without interference from others (especially parents), often viewing this interference with annoyance (quote 6). However, in many cases, the motivation was not only to avoid parental interference, but also worry (quote 7). In a different approach, one participant misreported SMBG to their treatment team in an effort to obtain longer intervals between clinic visits (quote 8).
  Participants were asked about the impact that embarrassment or stigma played on their testing habits and whether this led to misreporting. Responses varied, with some stating this had never been a problem, to others that would never test at school for this reason, leading to daily school related misreporting. Embarrassment, fear of being bullied, and wanting to avoid explaining diabetes to school peers (quote 9) were common motivations. Embarrassment stemming from revealing sub-optimal BGLs in front of others with diabetes at diabetes camp was another scenario reported (quote 10).
  Wanting to continue with an activity uninterrupted was a significant motivation for misreporting. In this instance, many said they were tempted to make up a number either verbally or in a logbook/pump so as to avoid the interruption caused by having to test or explain a suboptimal reading. This was reported across a variety of situations including being busy (quote 12), and being with friends (quote 11).

3) Avoidance of worry/concern in others or self
  Misreporting in order to avoid distress/worry in others was commonly reported. Participants felt that reporting a “bad” reading would make parents or doctors/nurses worried, disappointed, or frustrated, so misreported in an effort to avoid these reactions. This occurred both for logbook/pump downloads (quote 13) and verbal SMBG (quote 14). In combination with the theme above, participants also misreported in order to maintain their treatment independence, which they felt would be compromised if parents were worried about them (quote 12).
  Participants were also asked how their misreporting habits changed when they were upset/angry. Frustration or disappointment at suboptimal measurements, bad moods associated with high blood glucose, and feeling overwhelmed at the responsibility of treatment self-management were all reported as specific circumstances (quotes 15, 16, 17).

4) Ideas of how to decrease misreporting
  Finally, participants were asked what could be done to support them and reduce misreporting. Three main ideas emerged: modifying parental/diabetes team/others reactions to suboptimal blood glucose control, particularly in order to reduce the perception of negativity, anger, or judgement; using technology to bypass logbooks/records; and having parents/diabetes teams check their records more frequently for misreporting. In regard to this final point, it should be noted that feelings were polarised, with some older participants (3/15), thinking this might actually be detrimental, due to the increased interference and annoyance that may result. Examples of these polarised opinions were exhibited by participants 3 and 12 respectively:

“If mum was more forceful with me… and if she actually checked my meter (I would do it less).” Participant 3

“That would annoy me so much, so that would not have worked for me.” Participant 12

Some participants suggested that changing the way others, particularly parents and their treatment team, reacted when their BGLs were suboptimal would help them to misreport less. In particular, reducing perceived disappointment or anger was suggested.

“Yeah I don’t think I would (misreport) at all. Well, it’s not really frustration. You just kind of get the feeling that they’re disappointed in you, and disappointed that you haven’t been doing it. If they weren’t disappointed, maybe they should be, but I don’t think I’d lie.” Participant 6

## Discussion

While some of these themes are common sense, or previously speculated upon, this is the first study to actually examine and document the motivations behind misreporting blood glucose values in patients with T1DM. Clarifying this is important, as SMBG, and its reporting in various forms, remains an essential aspect of modern diabetes management. This is demonstrated by our finding of misreporting behaviour spanning not only traditional written logbook, but also verbal fabrications, and efforts to interfere with the accuracy of electronic pump and meter downloads. Due to the age of past publications in this area, electronic/pump misreporting has not previously been described. Additional key findings are the identified themes exploring this behaviour, including the common themes of achieving some benefit from misreporting, avoiding negative consequences from reporting suboptimal blood glucose, and avoiding worry/concern in others/self.

One overarching theme was misreporting to avoid negative consequences. This is consistent with the wider literature on the impact of negative consequences on diabetes treatment adherence. In particular, the observation that positive parental behaviours promote better adherence, while negative parental behaviours such as criticism or disciplinary action reduce adherence [12–14]. Stigma and social desirability have also been shown to affect treatment adherence in adolescents, with the presence of friends providing either positive or negative effects on adherence [15], while a strong desire to fit in may negatively affect adherence [16]. The polarised responses from our participants’ reflect and support these findings. Another key theme was avoidance of worry/concern, either in the participant themselves or in others. If their blood glucose was upsetting them, participants were more likely to misreport. In addition, if they felt that suboptimal blood glucose levels would upset their parents, misreporting occurred in an attempt to protect, or spare them worry or concern. Clearly, the way that adolescents with diabetes view themselves and the world around them can precipitate stress, resulting in reduced adherence [17]. Finally, misreporting occurred in circumstances where there was a perceived benefit. Most commonly, this occurred at school, and also by fabricating hypoglycaemia to access food. Some participants had the view that they deserved to take advantage of their illness sometimes and that “there’s got to be some perks about it” (participant 11). This manipulation of school situations has been previously described [18].

These adolescents all use reporting and misreporting as a functional way of manipulating their situation, either to achieve what they want, or to avoid what they do not want. Presenting oneself in a certain way in order to achieve a favourable outcome is a well-documented strategy in psychology and related disciplines [19]. One obvious way to reduce this behaviour would be to decrease the circumstances where misreporting is functional. An example in our findings is misreporting to prevent a negative reaction to the blood glucose reading, either anger, concern or frustration. Positively modifying this perception may provide an opportunity to reduce misreporting, as well as improve wellbeing.

As always with qualitative studies of this type, sample size is a limitation, and while we cannot claim that the results of this study include all possible themes and motivations leading to misreporting, the themes identified were generally consistent among the 15 participants, and no new themes/subthemes were raised in the final 3 interviews. Further exploration of this issue in other settings and cultures is required. In addition, some factors may have been missed due to reluctance on the part of participants to talk about some aspects of misreporting for fear of getting in trouble. Separating the interview from the treatment team and family was aimed at minimising this.

## Conclusion

Despite developments in technology, the reporting and self-monitoring of blood glucose remains an integral part of modern diabetes management. Misreporting of SMBG remains an important non-adherence behaviour, with patient techniques adapting to advances in technology. This study provides deeper insight into the motivations leading to this behaviour in adolescents, suggesting that further understanding and attention to this aspect of adherence may lead to improvements not only in glycaemic control and safety, but also to the psychological wellbeing of those with type 1 diabetes.

## Abbreviations

BGL, Blood glucose level; MDI, Multiple daily injections; NZDep2006, New Zealand Deprivation Index 2006; SMBG, Self-monitoring of blood glucose; T1DM, Type 1 diabetes
